# The Incidence of Depression among the Population of Central Kazakhstan and Its Relationship with Sociodemographic Characteristics

**DOI:** 10.1155/2017/2584187

**Published:** 2017-10-25

**Authors:** L. Turgunova, Ye Laryushina, A. Turmukhambetova, B. Koichubekov, M. Sorokina, I. Korshukov

**Affiliations:** Karaganda State Medical University, Gogol St. 40, Karaganda 100008, Kazakhstan

## Abstract

It has been established that the presence of depression is accompanied by an increased risk of morbidity and mortality in cerebrovascular and cardiovascular diseases and diabetes. The aim of this research was to estimate depressive symptom prevalence among the population in Central Kazakhstan and to define the relationship with social-demographic and behavioral factors. 1820 respondents of the population of Central Kazakhstan, aged 25 to 65, were performed. Participants included 777 urban and 1043 rural residents. Depressive symptoms assessed with the Patient Health Questionnaire (PHQ-9). The results showed that some degree of depressive symptoms was detected in 75.7% of the respondents. A minimal degree of depressive symptoms was observed in 28.51%, mild in 27.7%, moderate in 13.7%, and severe and very severe degree of depressive symptoms in 4.6% and 1.2%, respectively; the absence of depression symptoms was reported in 24.3% of the respondents. The study found a relationship between the prevalence of depressive symptoms and factors such as gender, education, income, presence of chronic diseases, and physical activity. We have not found a correlation between the frequencies of depressive symptoms with age, employment, character of labor, and marital status.

## 1. Introduction

Depression is an urgent medical and social problem due to its significant contribution to the global disease burden, characterising the health of the population. According to the WHO's forecast, by 2020, among the reasons for the loss of healthy life years due to temporary disability, disability, and early death (index of DALY), depression will be the second highest in all age groups [1]. Currently, depression already ranks second among the reasons for health loss in the age group 15–44 years. The development of severe depression is associated with an increase in the amount of suicidal ideation [2] and deterioration in the quality of life compared with the general population [3]. It has been established that the presence of depression is accompanied by an increased risk of morbidity and mortality in cerebrovascular [4] and cardiovascular diseases and diabetes [5, 6].

The prevalence of depression and its relationship with sociodemographic characteristics such as gender, age, marital status, income level, and education has been studied in various countries [7]. However, the frequency and degree of depression in the post-Soviet countries (including Kazakhstan) have been insufficiently studied. In addition, there are certain difficulties in conducting comparative analyses of the frequency of depression with those of other countries because the authors use different methods for studying depression. According to the territorial proximity, the level of socioeconomic development of Kazakhstan is mostly similar to that of Russia. The results of clinical and epidemiological studies, termed “compass,” conducted in 36 Russian cities showed that depressive spectrum disorders (nine or more points on the CES-D scale) were detected in 45.9% of patients; the percentage of respondents with a depressive state (total CES-D score of 26 points or more) was 23.8% [8]. In another study, the prevalence of depression in Russia (Novosibirsk) on the CES-D scale was 43.9% [9].

Some results of depression prevalence in other countries where the studies were conducted using the PHQ-9 were presented before. For example, in Saudi Arabia, the frequency of depression, defined with the PHQ-9, was 59% [10, 11], the urban population of Pakistan was 32.9% and that of Nigeria was 44.5%, and the prevalence was 20.1% in the United States [12]. The rate of mild depression was 14.8%, moderate depression was 4.5%, severe depression was 1.8%, and the rate of extremely severe depression was 0.6%. Despite the great interest in the study of depression, the researchers note that the number of patients with depression observed by primary healthcare doctors does not reflect the true prevalence of depression, as a significant proportion of patients with depression do not seek medical advice and remain undiagnosed [13].

Kazakhstan spreads across a very large territory while the population density is extremely low (6.51 people per square km). At the same time, different regions of the country (central, northern, eastern, southern, and western) differ by the level of the socioeconomic development, population density, climate conditions, and urbanization degree. In view of this, it is reasonable to expect different prevalence of depressive states depending on the place of residence.

Central Kazakhstan is an economic and geographical area with approximately 1,400,000 residents. It is an industrial region; the economy of which is based on the ferrous and nonferrous metallurgy, coal industry, mechanical engineering, and livestock breeding. The settlements are represented by 16 cities and 8 rural districts. The most common ethnic groups of the population are the Kazakhs and Russians, while the presence of other ethnic groups is also noticeable (Ukrainians, Koreans, etc.).

The purpose of our study was to perform research to estimate the depressive symptom prevalence among the population in Central Kazakhstan and to define the interdependence with social-demographic and behavioral factors. The results of this research could serve the basis for a larger scale research on the prevalence of depressive states in Kazakhstan as a whole.

## 2. Materials and Methods

### 2.1. Procedures

The study was conducted as part of the Scientific and Research Program that was supported by the Kazakhstan Republic Ministry of Healthcare (BP013). The research team consists of teachers from the Internal Medicine and Nutrition Hygiene Departments of Karaganda State Medical University, in addition to those with Master's degrees, residents, and interns. The research included a survey, for which the questionnaire for the participants of the study was developed.

Each participant was given a survey pack, which includes information for the participant, informed consent form, and the questionnaire itself. In the questionnaire, information on gender, age, social factors (income, living conditions, marital status, level of education, employment, and the nature of work), the presence or absence of chronic diseases, and the presence of physical activity was collected. A question about physical activity was formulated as “Do you usually have daily at least 30 minutes physical activity at work and/or during leisure time including normal daily activity? (Yes/no).”

Depressive symptoms were assessed by the Russian version of the Patient Health Questionnaire (PHQ-9), which had previously been checked for sensitivity and specificity [14]. Historically, the situation is that the indigenous population, as well as other non-Russian residents of Central Kazakhstan, is fluent in Russian.

The PHQ-9 is a brief patient self-report depression assessment tool that was derived from the interview-based PRIME-MD [15]. It was specifically developed for use, in primary care general medical settings. The PHQ-9 offers several advantages to other tools. The tool is easily understood with very high face validity for patients and clinicians in primary care, because the items and the scoring of items on the PHQ-9 are identical to the symptoms and signs of DSM-4 major depression. Many other instruments use a 1-week time frame, but the PHQ-9 uses a 2-week time frame, which conforms to DSM-4 criteria. It is the only tool that was specifically developed for use as a patient self-administered depression diagnostic tool, rather than as a severity or screening tool. It is the only short self-report tool that can reasonably be used both for the diagnosis of DSM-4 major depression and for the tracking of the severity of major depression over time. Psychometric evaluation of the PHQ-9 revealed a sensitivity ranging from 62%–92% and a specificity between 74% and 88%.

The questionnaire consists of nine points with a four-point scale evaluation (absence of symptoms, some days, more than half the days, and almost every day), allowing for a diagnosis of depression occurring in the previous two weeks to be made for those scoring in the range of 0–27 points. The sum of points from 1–4 was regarded as minimal depressive symptom severity, 5–9 mild, 10–14 moderate, 15–19 severe, and 20–27 extremely severe depressive symptom.

### 2.2. Subjects

From a public health department of two cities and two rural districts of the Karaganda Region, general registries were obtained. To make a random sample from those registries, every second record was taken. Invitations for those people were sent by phone. During two weeks, 2167 respondents arrived to surveying. Each of the possible participants was informed by the research team. Of these, 1820 of the respondents, aged 25 to 65, gave informed consent to participate in the research and were fluent in Russian; these individuals were included in the study. Among them, 777 were living in urban areas and 1043 in rural. The exclusion criteria included being pregnant or having a mental or severe neurological disease. A total of 347 people were excluded from the study due to different reasons: they interrupted the survey, gave incomplete answers to the questionnaire, lacked time, or lacked interest in the study.

### 2.3. Statistical Methods

We used cross-tabulations to assess the prevalence of depressive symptoms in different sociodemographic groups. The number of participants in each subgroup was counted, in addition to the percentage of the total sample and 95% confidence intervals (95% CI). The statistical significance of differences was assessed using a chi-square test.

The relative risk of the depressive symptoms under the influence of social-demographic factors was evaluated by ordinal logistic regression. The significance of the impact from each attribute on the level of depressive symptoms was assessed using the Wald test. Logistic regression coefficients and the impact of attributes on the probability of depressive symptom level in the form of odds ratios (OR) were calculated. Odds ratios were evaluated relative to a reference group.

## 3. Results and Discussion

General characteristics of contingent subjects are presented in Table 1.

The results showed that 519 (28.5%) demonstrated the minimum degree of depressive symptoms, 504 (27.7%) had mild, 250 (13.7%) exhibited symptoms of moderate degree, and 83 (4.6%) and 22 (1.2%) of cases showed severe and very severe depressive symptoms; the absence depression symptoms were reported in 442 (24.3%) of respondents. Depressive symptomatology frequencies according to sociodemographic and behavioral factors are presented in Table 2.

### 3.1. Depressive Symptoms and Gender

29.6% of men and 19.7% of women showed no signs of depressive symptoms. As the depressive symptoms increase, the gender differences reduce: very severe depressive symptoms were observed in 1.2% of women and 1.1% of men.

### 3.2. Depressive Symptoms and Age

There were no significant differences in the prevalence of depressive symptoms depending on age; although the highest percentage of persons who had no depressive symptomatology was observed in the age group 36–45 years (26.9%), the highest percentage of people with very severe degree was in the age group over 60 years (2.2%).

### 3.3. Depressive Symptoms and Ethnic Origin

In our study, 62% of the respondents were persons belonging to the Kazakh ethnic group; among them, the frequency of depressive symptoms was significantly lower compared to the respondents who identified themselves as belonging to the Russian or other ethnic groups.

### 3.4. Depressive Symptoms and the Marital Status

The lack of depressive symptoms was more frequently registered among persons who had not married or those who were then married (24.9%); severe or very severe degree was observed among those who were divorced (8.2% and 3%, resp.), as well as among widowed persons (6.5% and 2.4%, resp.).

### 3.5. Depressive Symptoms and Education

Although the results showed that differences in the prevalence of depressive symptoms depend on the level of education, this relationship was nonlinear. The lowest frequency of moderate, severe, and very severe depressive symptomatology cases was observed in persons with higher levels of education.

### 3.6. Depressive Symptoms, Employment, and the Nature of Labor

Differences were noted in the incidence of depressive symptomatology, depending on the nature of employment and labor. A lower frequency of severe depressive symptoms was found among the respondents who have studied/worked and those who performed mental labor.

### 3.7. Depressive Symptoms and Income

The rate of depressive symptoms existed to varying degrees among groups of respondents with different income levels. Among the respondents who declared having an “average,” “above average,” or “high” income level, there appears to be a lower incidence of severe and very severe degree of depressive symptoms.

### 3.8. Depressive Symptoms and Chronic Illness

The presence of a chronic disease increases the incidence of depressive symptomatology, regardless of its severity.

### 3.9. Depressive Symptoms and Physical Activity

The frequency of depressive symptoms had a significant difference among those who did and did not engage in physical activity.

Table 3 illustrates the results of the attribute evaluation using an ordinal logistic regression. According to these data, in females, the probability of depressive symptoms in 1.43 (95% CI: 1,2; 1,7) is higher than that in men. The OR in persons of Russian nationality compared with Kazakh is 1.59 (95% CI: 1.29; 1.94), and in other nationalities, OR = 1.62 (95% CI: 1.27; 2.08). Also, depressive symptom risk factors include financial income below average, low physical activity, and the presence of chronic diseases. In the obtained model of ordinal regression, all these factors turned out to be statistically significant. It should be noted that the risk of depressive symptoms in workers (OR = 2.35) (95% CI: 1.15; 4.80) and in the unemployed is higher (OR = 2.52) (95% CI: 1.22; 5.2) than that in the students.

As noted above, we did not receive statistically significant differences in the prevalence of depressive symptoms in different age groups. The results of regression analysis also indicate that age is not a risk factor (*p* > 0.05).

We have not found any information on the depressive symptom prevalence in Kazakhstan in any available literature. In the current study, a higher rate of depressive symptomatology was observed in women compared to men, which has been supported by many studies. It can be assumed that the differences according to sex are due to the greater willingness of women to discuss their psychological problems and a more positive attitude in general to mental disorders compared to the tendencies of men [16–18]. There are prejudices that exist related to the stigma associated with depression, and these may be less important for women than for men [19]; thus, women are more willing to accept the presence of depression [20–22]. Lower levels of bias and prejudice against the presence of depressive symptoms among women may explain the greater incidence of depression compared with men.

Health and physical activity are important factors that have an impact on depressive symptomatology. We revealed the fact that among persons with chronic diseases, depressive symptoms are more common, than among the healthy people. These data are consistent with other studies that have shown that the relationship between depression and chronic diseases illustrates a mutually burdening character. The presence of depression, in turn, significantly aggravates the clinical course of internal diseases [23, 24], complicates rehabilitation and secondary prevention, increases the cost of treatment, impairs the quality of life, and increases the risk of morbidity and mortality in patients with chronic diseases [25]. Among physically active persons, the percentage of people who have no depressive symptoms is higher and the percentage of those who have high or very high depression level is lower.

The above results are consistent with many recorded data for other countries. However, there have been revealed some peculiar features probably specific of Kazakhstan only due to its unique socioeconomic and cultural traditions.

We did not find the dependency of depressive symptom prevalence based on age. The relationship between depressive symptomatology and age varies greatly in different studies. In general, the relationship between age and depression was significantly greater in high-income countries compared to low- and middle-income countries. In high-income countries, the risk of depression was associated with young age; in other countries, the prevalence of depression was higher in the elderly. Our results can be associated with the fact that in Kazakhstan many elderly and senior citizens reside with their adult children's families, which improves the quality of life of this population group.

In most studies, marital status is seen as being a significant factor in the prevalence of depression [16, 22, 26]. The divorced, widow/widowers, and people who have never been married are prone to the risk of depression. In our study, we found no significant relationship with marital status, possibly due to the fact that 75.9% of respondents were married. In addition, persons over the age of 65 were not included in the study; among the ageing population, the percentage of single people is much higher, given the life expectancy in Kazakhstan in 2013 of 65.8 years among men and 75.1 years among women.

The level of education and income of the respondents significantly influenced the prevalence of depressive symptoms. According to a meta-analysis [22], the level of income was a significant factor in the development of severe depression in countries with a high level of development and had no significant association in countries with lower income levels. In general, the relationship between depression and the level of education did not differ between countries with different income levels. In our study, dependence from an educational level was nonlinear. The risk of depressive symptoms was lower among respondents with a higher level of education; the greatest risk of minimum, moderate, and severe major degree of depressive symptoms was associated with secondary special education. This issue requires in-depth study of the characteristics of the group of respondents that participated in secondary special education.

## 4. Conclusion

Thus, the findings emphasise the importance of the depression symptom prevalence problem in Kazakhstan and allow for the identification of groups that have the greatest risk of developing depressive symptomatology. However, these results are preliminary and can serve the basis for further research of depressive states among the Kazakhstan population with a statistical evaluation of the results. There is a need to study the prevalence of depressive symptoms in other regions of the Republic, including in the urban and rural population belonging to the age group over 65 years. The data on the impact of the age and marital status should also be specified.

## Figures and Tables

**Table 1:** General characteristics of the contingent.

Attributes	*N*	%
Gender		
Female	974	53.5
Male	846	46.5

Age		
25–35 yrs	479	26.3
36–45 yrs	361	19.8
46–60 yrs	756	41.5
61–65 yrs	224	12.2

Nationality		
Kazakhs	1119	61.5
Russians	441	24.2
Other	260	14.3

Family status		
Married	1341	73.7
Single	221	12.1
Divorced	134	7.4
Widower	124	6.8

Chronic diseases		
No	785	43.1
Yes	1035	56.9

Physical activity		
No	316	17.4
Yes	1504	82.6

Employment		
Unemployed	624	34.3
Employed	1168	64.2
Learning	28	1.5

Education		
Below the medium	64	3.5
Medium	697	38.3
Specialized secondary	565	31.0
Higher education	494	27.1

Type of work		
Brainwork	690	37.9
Physical work	1130	62.1

Income		
Low	706	38.8
Below the average	239	13.1
Average	635	34.9
Above average	224	12.3
High	16	0.9

**Table 2:** Prevalence of depressive symptoms in different sociodemographic groups.

Attributes	*N*	0	1 (1–4)	2 (5–9)	3 (10–14)	4 (15–19)	5 (20–27)	*p* level
		*n*/*p*% (95% CI)	*n*/*p*% (95% CI)	*n*/*p*% (95% CI)	*n*/*p*% (95% CI)	*n*/*p*% (95% CI)	*n*/*p*% (95% CI)	
*Gender*								
Female	967	189/19.7 (17.22; 22.18)	276/28.53 (25.71; 31.35)	285/29.44 (26.59; 32.29)	158/16.35 (14.04; 18.66)	47/4.77 (3.44; 6.1)	12/1.22 (0.53; 1.91)	0.000^∗^
Male	853	254/29.78 (26.71; 32.85)	244/28.6 (25.57; 31.63)	219/25.67 (22.74; 28.6)	90/10.55 (8.49; 12.61)	36/4.22 (2.87; 5.57)	10/1.17 (0.45; 1.89)	
*Nationality*								
Kazakhs	1128	316/28.01 (25.39; 30.63)	336/29.79 (27.12; 32.46)	299/26.51 (23.93; 29.09)	129/11.44 (9.58; 13.3)	42/3.72 (2.62; 4.82)	6/0.53 (0.11; 0.95)	0.000^∗^
Russians	447	82/18.34 (14.75; 21.93)	119/26.62 (22.52; 30.72)	136/30.43 (26.16; 34.7)	73/16.33 (12.9; 19.76)	30/6.71 (4.39; 9.03)	7/1.57 (0.42; 2.72)	
Other	245	44/17.96 (13.15; 22.77)	63/25.71 (20.24; 31.18)	72/29.39 (23.69; 35.09)	47/19.18 (14.25; 24.11)	10/4.08 (1.6; 6.56)	9/3.67 (1.32; 6.02)	
*Education*								
Elementary	64	14/21.88 (11.75; 32.01)	20/31.25 (19.89; 42.61)	13/20.31 (10.45; 30.17)	11/17.19 (7.95; 26.43)	5/7.81 (1.24; 14.38)	1/1.56 (0; 4.6)	0.000^∗^
Secondary	702	204/29.06 (25.7; 32.42)	177/25.21 (22; 28.42)	181/25.78 (22.54; 29.02)	93/13.25 (10.74; 15.76)	39/5.56 (3.86; 7.26)	8/1.14 (0.35; 1.93)	
Secondary special	557	101/17.72 (14.59; 20.85)	172/31.05 (27.25; 34.85)	168/28.42 (24.72; 32.12)	92/16.67 (13.61; 19.73)	23/4.04 (2.42; 5.66)	12/2.11 (0.93; 3.29)	
Higher	497	128/25.75 (21.91; 29.59)	151/30.38 (26.34; 34.42)	151/30.38 (26.34; 34.42)	51/10.26 (7.59; 12.93)	15/3.02 (1.52; 4.52)	1/0.2 (0; 0.59)	
*Employment*								
Unemployed	624	143/22.92 (19.62; 26.22)	163/26.12 (22.67; 29.57)	166/26.6 (23.13; 30.07)	101/16.19 (13.3; 19.08)	40/6.41 (4.49; 8.33)	11/1.76 (0.73; 2.79)	
Employed	1168	289/24.74 (22.27; 27.21)	345/29.54 (26.92; 32.16)	332/28.42 (25.83; 31.01)	148/12.67 (10.76; 14.58)	43/3.68 (2.6; 4.76)	11/0.94 (0.39; 1.49)	0.014^∗^
Learning	28	10/35.71 (17.96; 53.46)	11/39.29 (21.2; 57.38)	6/21.43 (6.23; 36.63)	1/3.57 (0; 10.44)	0/0 (0; 0)	0/0 (0; 0)	
*Type of work*								
Brainwork	695	153/22.01 (18.93; 25.09)	228/32.81 (29.32; 36.3)	196/28.2 (24.85; 31.55)	87/12.52 (10.06; 14.98)	25/3.6 (2.21; 4.99)	6/0.86 (0.17; 1.55)	0.003^∗^
Physical work	998	245/24.55 (21.88; 27.22)	247/24.75 (22.07; 27.43)	283/28.36 (25.56; 31.16)	152/15.23 (13; 17.46)	55/5.51 (4.09; 6.93)	16/1.6 (0.82; 2.38)	
*Income*								
Low	650	144/22.33 (19; 25.66)	166/26 (22.49; 29.51)	180/28.33 (24.72; 31.94)	110/16.67 (13.69; 19.65)	42/5.33 (3.53; 7.13)	8/1.33 (0.41; 2.25)	0.000^∗^
Below the average	236	33/13.98 (9.56; 18.4)	76/32.2 (26.24; 38.16)	66/27.97 (22.24; 33.7)	37/15.68 (11.04; 20.32)	17/7.2 (3.9; 10.5)	7/2.97 (0.8; 5.14)	
Average	686	189/28.14 (24.65; 31.63)	183/27.2 (23.74; 30.66)	201/30.03 (26.47; 33.59)	82/11.32 (8.86; 13.78)	26/2.52 (1.3; 3.74)	5/0.79 (0.1; 1.48)	
Above average	232	49/21.78 (16.39; 27.17)	73/32.44 (26.32; 38.56)	54/24 (18.42; 29.58)	36/16 (11.21; 20.79)	12/5.33 (2.39; 8.27)	1/0.44 (0; 1.3)	
High	16	4/25 (3.78; 46.22)	8/50 (25.5; 74.5)	3/18.75 (0; 37.88)	1/6.25 (0; 18.11)	0/0 (0; 0)	0/0 (0; 0)	
*Chronic diseases*								
No	782	235/30.05 (26.84; 33.26)	225/28.77 (25.6; 31.94)	200/25.58 (22.52; 28.64)	88/11.25 (9.04; 13.46)	31/3.96 (2.59; 5.33)	3/0.38 (0; 0.81)	0.000^∗^
Yes	1038	206/19.85 (17.42; 22.28)	297/28.61 (25.86; 31.36)	304/29.29 (26.52; 32.06)	160/15.41 (13.21; 17.61)	52/5.01 (3.68; 6.34)	19/1.83 (1.01; 2.65)	
*Physical activity*								
No	321	81/25.48 (20.66; 30.3)	106/33.12 (27.91; 38.33)	83/25.8 (20.96; 30.64)	27/8.28 (5.23; 11.33)	17/5.1 (2.67; 7.53)	7/2.23 (0.6; 3.86)	0.008
Yes	1499	349/23.28 (21.14; 25.42)	418/27.89 (25.62; 30.16)	425/28.35 (26.07; 30.63)	225/15.01 (13.2; 16.82)	67/4.47 (3.42; 5.52)	15/1 (0.5; 1.5)	

^∗^Statistically significant differences (chi-square test, *p* < 0.05).

**Table 3:** Estimation of ordinal logistic regression parameters.

	Estimate	St. Er	Wald	Level	OR	95% CI	
						Lower	Upper
Threshold							
[Depressive symptoms = minimal]	0.54	0.61	0.80	0.370	1.73	0.52	5.73
[Depressive symptoms = mild]	1.86	0.61	9.21	0.002	6.42	1.93	21.37
[Depressive symptoms = moderate]	3.22	0.61	27.48	0.000	25.20	7.55	84.27
[Depressive symptoms = moderately severe]	4.63	0.62	55.64	0.000	103.34	30.54	349.67
[Depressive symptoms = severe]	6.25	0.65	92.68	0.000	522.70	146.20	1868.70
Location							
[Gender = female]	0.35	0.08	15.87	0.000	1.43	1.20	1.70
[Gender = male]	0^a^	.	.	.			
[Age = 24–35]	0.12	0.16	0.61	0.434	1.13	0.83	1.55
[Age = 36–45]	−0.15	0.16	0.87	0.349	0.86	0.62	1.18
[Age = 46–60]	−0.05	0.14	0.11	0.732	0.95	0.72	1.27
[Age = 61–65]	0^a^	.	.	.			
[Nationality = other]	0.48	0.12	14.62	0.000	1.62	1.27	2.08
[Nationality = Russian]	0.46	0.10	19.55	0.000	1.59	1.29	1.94
[Nationality = Kazakhs]	0^a^	.	.	.			
[Education = elementary]	0.15	0.25	0.37	0.542	1.17	0.71	1.92
[Education = secondary]	−0.05	0.12	0.20	0.650	0.94	0.74	1.21
[Education = secondary special]	0.20	0.12	3.00	0.083	1.23	0.97	1.56
[Education = higher]	0^a^	.	.	.			
[Employment = unemployed]	0.92	0.37	6.19	0.013	2.52	1.22	5.20
[Employment = employed]	0.85	0.36	5.47	0.019	2.35	1.15	4.80
[Employment = learning]	0^a^	.	.	.			
[Work type = brainwork]	−0.03	0.10	0.13	0.711	0.96	0.78	1.18
[Work type = physical work]	0^a^	.	.	.			
[Income = low]	0.73	0.46	2.50	0.114	2.08	0.84	5.14
[Income = below the average]	1.08	0,472	5.269	0.022	2.95	1.17	7.45
[Income = average]	0.56	0.462	1.472	0.225	1.75	0.71	4.33
[Income = above average]	0.83	0.473	3.145	0.076	2.31	0.92	5.84
[Income = high]	0^a^	.	.	.			
[Chronic diseases = no]	−0.42	0.091	22.471	0.000	0.65	0.54	0.78
[Chronic diseases = yes]	0^a^	.	.	.			
[Physical activity = no]	0.27	0.111	6.103	0.013	1.32	1.06	1.64
[Physical activity = yes]	0^a^	.	.	.			

Link function: logit. ^a^This parameter is set to zero because it is redundant.
